# Supplementary material for: Venom Function of a New Species of Megalomyrmex Forel, 1885 (Hymenoptera: Formicidae)
Source: Toxins (Basel). 2020 Oct 29;12(11):679. doi: 10.3390/toxins12110679 (PMC7693960; doi:10.3390/toxins12110679)
Supplement: Supplementary file 1 [file toxins-12-00679-s001.pdf]

Supplementary Materials: Venom Function of a New Species of *Megalomyrmex* Forel, 1885 (Hymenoptera: Formicidae)

Kyle Sozanski, Lívia Pires do Prado, Andrew J. Mularo, Victoria A. Sadowski, Tappey H. Jones and Rachelle M.M. Adams

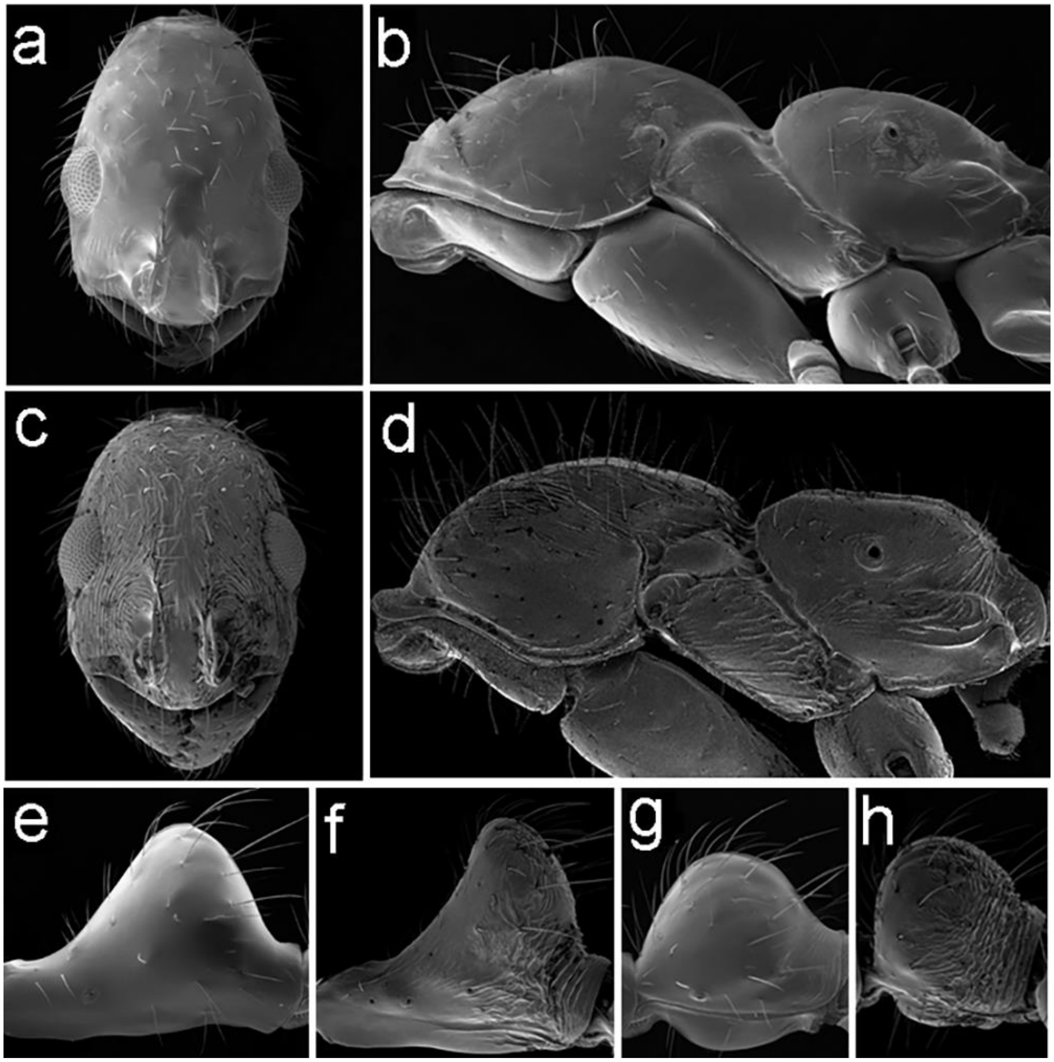

Figure S1. Scanning electron micrographs highlighting sculpturing differences between workers of *Megalomyrmex peetersi* sp. n. (Colombia) and *Megalomyrmex wallacei* (Brazil). (**a**) Head in frontal view (*M. peetersi*), (**b**) mesosoma in lateral view (*M. peetersi*), (**c**) head in frontal view (*M. wallacei*), (**d**) mesosoma in lateral view (*M. wallacei*), (**e**) petiole in lateral view (*M. peetersi*), (**f**) petiole in lateral view (*M. wallacei*), (**g**) postpetiole in lateral view (*M. peetersi*) and, (**h**) postpetiole in lateral view (*M. wallacei*).
